# Equivalent Circuit-Assisted Multi-Objective Particle Swarm Optimization for Accelerated Reverse Design of Multi-Layer Frequency Selective Surface

**DOI:** 10.3390/nano12213846

**Published:** 2022-10-31

**Authors:** Yaxi Pan, Jian Dong, Meng Wang

**Affiliations:** School of Computer Science and Engineering, Central South University, Changsha 410083, China

**Keywords:** frequency selective surface, equivalent circuit, optimization, building blocks, semi-empirical formula

## Abstract

In this paper, a fast reverse design method of multi-layer frequency selective surface (FSS) based on the equivalent circuit (EC)-assisted multi-objective particle swarm optimization (MOPSO) algorithm is proposed. Converting the desired frequency response requirements into an EC and then determining structural parameters via building blocks’ EC and MOPSO simplifies the inverse design process of the FSS. The layer-by-layer building blocks of EC are used instead when dealing with the problem of complicated EC computation associated with multi-layer FSS. By converting factors that are difficult to calculate, such as interlayer coupling, into an MOPSO seeking process, the computational complexity is reduced while the design accuracy can be improved. To begin with, it is necessary to determine the distribution of zeros and poles according to the design goals in order to calculate the appropriate EC. Then, the preliminary design of the FSS has been completed in accordance with the EC and the associated building block structure. Finally, the objective function of the optimization algorithm is determined according to the desired frequency response, and the FSS structure parameters are optimized. Taking dual band-stop FSS and triple band-pass FSS structures as examples, the transmission coefficient results obtained by the proposed reverse design method are consistent with the transmission coefficient results based on the ECs, which verifies the effectiveness of the proposed method. The optimized triple band-pass FSS demonstrates strong stability even at oblique incident angles of up to 45° in both TE and TM polarizations.

## 1. Introduction

The frequency selective surface (FSS) has promising applications in the microwave field as a periodic structure with spatial and frequency filtering properties [1,2,3,4,5,6]. The analysis of the FSS is usually performed by establishing the corresponding physical model after determining the parameters such as the form and size of the FSS and then calculating the reflection or transmission coefficient. The finite FSS unit, periodic method moments, and equivalent circuit (EC) are often used to analyze electromagnetic (EM) structures based on FSS [7,8,9,10,11]. To solve the obstacles of the sizeable computational volume and long optimization cycle required for the design of an FSS structure in conventional design methods, the EC came into being [12,13,14,15,16]. These methods calculate the equivalent inductance *L* and equivalent capacitance *C* of the FSS using an EC that depends on the geometry of the FSS [17,18]. Any change in the size of the FSS will cause a corresponding change in the inductance and capacitance values [19,20]. Therefore, the EC is simple, effective, and intuitive.

There are generally two fundamental analysis and solution methods for its performance characteristics for any microwave system composed of an infinite two-dimensional periodic structure, supporting and matching dielectric layers, and free space on both sides. One is based on the Floquet model theory, applying periodic boundary conditions and full-wave numerical simulation software, for instance, HFSS and CST [21,22]. It can numerically solve the EM field distribution in the microwave system accurately and then calculate the reflection, absorption and transmission, and other characteristics of the system EM waves. The other is to equate the microwave system to a microwave network and then use EC and microwave network theory to qualitatively and approximately analyze and solve the network parameters such as impedance *A*, and *S* [23]. The first method gives rigorous and accurate results, but it seldom involves analyzing the regulation principle of the periodic structure on the electromagnetic wave. Generally, only the designed structure can be analyzed and solved. The latter one allows a qualitative analysis of the network characteristics and operating principles of the FSS. It can also serve as guidance for the design and performance optimization of the FSS, but it cannot obtain details such as the internal field and current system distribution. Since the EC can directly reflect the filtering characteristics of the periodic structure, many researchers have tried to derive an exact formula that can fully reproduce the traits of FSS [24].

In short, these proposed formulas can approximate the quasi-static model of a simple FSS. However, these formulas are often complex and inaccurate for complex FSS models. Amir did not directly apply the empirical formula when designing the square loops FSS in [25]. Instead, he simplified some widely used EC-based analytical formulations and validated the relationship between the square loops FSS and the EC elements. According to the requirements, the corresponding structure parameters were selected from the curves for simulation verification. This method can guide the simple FSS design and often needs to be revised to ensure accuracy. On behalf of expanding the scope of the application of an equivalent circuit model (ECM), Fllipo edited the circuit lumped parameters of a single-layer FSS in [26] and extended it to the analysis of a multi-layer FSS cascade using the method of transmission matrix multiplication. It can be used to calculate the reflection coefficient and transmission coefficient curve of FSS at various frequencies and predict FSS performance more quickly. The larger the number of FSS layers in the design, the greater the amount of calculation for the transmission matrix multiplication. The analysis of matrix multiplication does not consider the influence between different layers of FSS in practical applications. Therefore, the extraction method of EC parameters is still not accurate enough. In the design process, the coupling effect between the layers of the FSS is often ignored when using the method in reference [27].

In order to accurately and quickly design compliant FSSs, combining optimization algorithms with traditional design processes has become a research hotspot in recent years. A domain decomposition hybrid genetic algorithm (GA) incorporating a local optimization scheme for real-coded quantities has been applied to optimize FSS [28,29,30,31]. A unique development strategy that involved selecting the most suitable magnetic substrate from a GA database has been adopted [32]. The basic idea of optimization is to divide the pattern or substrate material, encode, and finally search and optimize to reach the design goal. In [33], Yilmaz integrated the particle swarm optimization (PSO) algorithm into the design of the square ring FSS and optimized the FSS structure size with its EC transfer function as the objective function. Because the transfer function selected here is only for a fixed EC, and there is no general method for extracting the transfer function of the FSS EC, the optimization algorithm using the transfer function as the objective function is not universal.

This paper proposes a design method to improve the design accuracy of the FSS that combines full-wave simulation software with EC and employs optimization algorithms as auxiliary means. First, we use the basic FSS building blocks corresponding to the EC for preliminary design aiming at the design goal. Then, the full-wave simulation and intelligent algorithm are applied to optimize the structure parameters of FSS. Taking the design of dual band-stop FSS and triple band-pass FSS as examples, the effectiveness of this method is verified. At the same time, we have processed and experimentally measured the triple band-pass FSS in this paper, and the results are consistent with the simulation results. This method does not pursue the derivation of accurate formulas such as multi-layer transmission matrix or modified EC transmission coefficients to calculate the correct structure parameters. Instead, factors that are not easy to calculate, such as the influence of the dielectric substrate, the impact of polarization, and the effect of coupling between multi-layer FSS, are transferred into the optimization algorithm to eliminate. Therefore, the applicability of this method is not limited to the FSS of a specific building block. Using the EC to design the rough structure and then the optimization algorithm to accurately design also reduces many calculation costs.

The rest of the paper is organized as follows. In Section 2, the reverse design method based on the ECM is proposed. An analysis of the FSS building blocks and their corresponding EC is presented, as well as a detailed description of how the proposed method is designed. Section 3 verifies the effectiveness of the proposed method through two design examples of dual band-stop and thriple band-pass FSS. Section 4 conslude the paper.

## 2. Reverse Design Method of FSS Based on the ECM

According to the existing research, many FSSs are designed to rely heavily on full-wave simulation, with parametric scanning playing a significant role in this process. Nevertheless, while numerical simulations produce an accurate frequency response for a given FSS structure, there is insufficient information about beginning the design of the FSS and how to initialize its geometry so that the expected frequency response is achieved. In addition to providing the designer with an approximation of the frequency response with an acceptable level of accuracy, the ECM is frequently used to determine how an FSS operates. However, it’s seldom found that FSS designs incorporate ECM as a design tool to achieve the desired frequency response.

The filter corresponds to an equivalent circuit consisting of four functions: low-pass, high-pass, band-pass, and band-stop. Consequently, a filter with arbitrary frequency response can be implemented. The FSS building blocks structure can realize these four filtering functions. In addition, since these block structures are symmetrical and have better polarization and oblique incidence angle insensitivity, they can be used as candidate structures for building more complex and more performance-demanding FSSs.

### 2.1. FSS Building Blocks and Their EC

**Figure 1:**
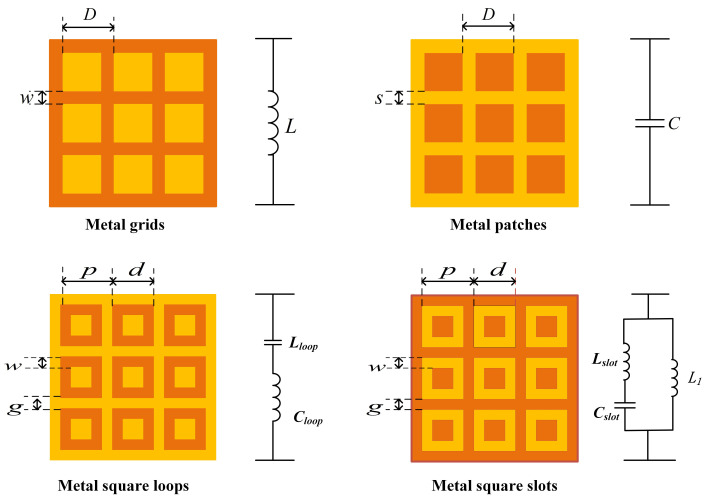
FSS building blocks and its EC.

Since the 1980s, E.A. Parker has analyzed the EC model of numerous building blocks of FSS, such as metal square slots and square loops [12]. The EM structure based on an FSS is similar to the filter in circuit theory, including a low-pass filter, high-pass filter, band-pass filter, and band-stop filter. Depending on the filtering characteristics, the EC of FSS can be represented as a series or parallel resonant RLC circuit (as shown in Figure 1). After correctly establishing the EC model, extracting effective EC parameters is the key to designing FSS using EC.

The equivalent inductance *L* for metal grids and the equivalent capacitance *C* for metal patches can be defined as [6]: The TM incidence occurs when the electric field polarization is parallel to the incidence plane, i.e., θ=0°, and TE incidence occurs when the electric field is perpendicular to the incidence plane, i.e., ϕ=0°. Therefore, the impedance expressions for TE and TM incidence are needed to simulate the array at oblique incidence angles. For the square loops structure, the semi-empirical relationship obtained by transmission line theory is defined as:

(1) $$\left\{\begin{array}{c} \frac{X_{Lloop}}{Z_{0}}=\omega L_{loop}=\frac{d}{p}F\left(P,2w,\lambda ,\theta \right),\\ \frac{B_{Cloop}}{Z_{0}}=\omega C_{loop}=\frac{d}{p}\cdot 4\varepsilon_{eff}F\left(P,g,\lambda ,\theta \right), \end{array}\right.$$

(2) $$F\left(P,2w,\lambda ,\Psi \right)=\frac{p}{\lambda }\left(ln\left(csc\frac{\pi w}{2p}\right)+G\left(P,w,\lambda ,\Psi \right)\right),$$

where G(P,w,λ,Ψ) is derived in [12]. If Ψ=θ, it indicates the case of TE polarization. Otherwise, if Ψ=ϕ, it indicates the case of TM polarization.

$X_{Lloop}$ is the normalized inductive reactance of the square patches and the slots, $B_{Cloop}$ is the normalized capacitive reactance, $Z_{0}$ is the characteristic impedance of the free space, *p* represents the length of the array period, *d* is the side length of the square ring, *w* is the width of the square loop side, *g* represents the slot between the square rings.

The EC of the metal slots is shown in Figure 1, including an inductor $L_{1}$ representing the grid, with a series resonant circuit $L_{slot}$ and $C_{slot}$ representing the square patch. Their values correspond to the parameters of the metal slots structure, which can be expressed as:

(3a) $$\begin{array}{cc} \frac{X_{L_{1}}}{Z_{0}} & =\omega L_{1}=F\left(p,w,\lambda ,\theta \right), \end{array}$$

(3b) $$\begin{array}{cc} \frac{X_{LS_{1}}}{Z_{0}} & =\frac{p-2g}{2p}F\left(p,d-2g,\lambda ,\theta \right), \end{array}$$

(4) $$\frac{X_{Lslot}}{Z_{0}}=\omega X_{Lslot}=X_{LS_{1}}+\frac{g}{d-2g+w}X_{L1},$$

(5a) $$\begin{array}{cc} \frac{B_{C1}}{Y_{0}} & =\omega C_{1}=4F\left(p,d,\lambda ,\theta \right), \end{array}$$

(5b) $$\begin{array}{cc} \frac{B_{C2}}{Y_{0}} & =\omega C_{2}=4F\left(d-g,g,\lambda ,\theta \right), \end{array}$$

(6) $$\frac{B_{Cslot}}{Y_{0}}=\omega C_{slot}=\left(1.75B_{C1}+0.6B_{C2}\right)\varepsilon_{eff}.$$

For a multi-layer FSS composed of an *n*-layer FSS and (n−1)-layer dielectric substrate, the transmission line model adopted by Flipo can be used to approximate the construction of the transmission matrix [18]. This method uses more complicated calculations and approximates the transmission matrix of a multi-layer FSS, which has good generality and versatility. Its detailed calculation process had been proved in [27]. Optimization algorithms are used to compensate for factors that increase the computational complexity, such as the effect of coupling between FSS layers. Therefore, the application of this method is not limited to the form of specific building blocks of the FSS.

### 2.2. Design Process of the Proposed Method

**Figure 2:**
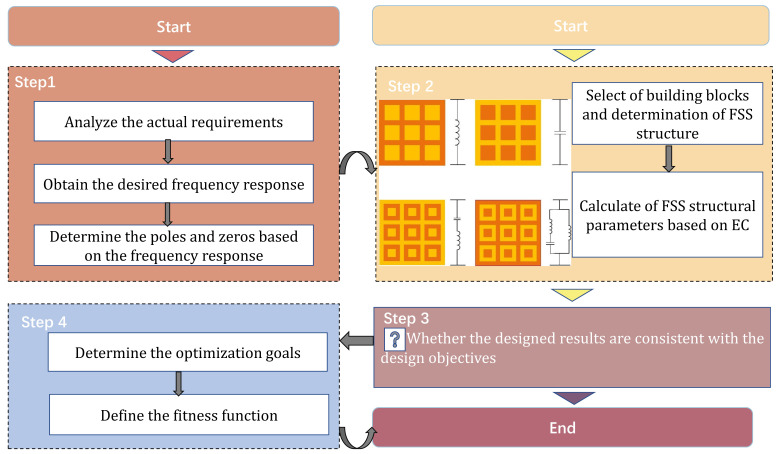
Flow chart of the proposed method.

The mode matching method, finite element method, spectral-domain method, etc. can calculate the EM response more accurately, but its calculation process is more complicated. So, a simple EC was proposed, which can design the FSS relatively quickly. The proposed method designs the FSS inversely by mapping the EC of simple building blocks to the EM field. The design procedure of the proposed method is shown in Figure 2. The design steps are briefly summarized as follows:

First, the design objectives are analyzed from the actual requirements. This distribution of zeros and poles is determined by the filtering characteristics (band-pass/band-stop) of the desired frequency response.

Second, the initial FSS design phase. From the distribution of zeros and poles in step 1, the topology of the EC and the values of its components L, C are determined. Then, the FSS topology is determined based on the FSS building block structure shown in Figure 1 with the corresponding equivalent circuit. Finally, the values of the FSS structure parameters are calculated according to Section 2.1.

Third, determine whether the initial designed FSS frequency response is consistent with the target. If it is not consistent, go to the next step.

Finally, the objective function of the multi-objective particle swarm optimization (MOPSO) algorithm is determined. The solution that satisfies the design objective is obtained in the Pareto solution of the MOPSO algorithm.

During the design process, there are a few points that need to be specifically stated:

(1) Instead of using a multi-layer FSS transmission matrix for the multi-layer FSS design, we use the basic layer-by-layer building blocks for the design, which reduces the computational complexity.
(2) We can obtain the size range of the FSS based on the operating frequency and wavelength of the resonant circuit. The resonant wavelength of the FSS roughly corresponds to the perimeter of the array cell, D≈λ/4.
(3) Considering that the dielectric loading will cause the center frequency drift, for the dielectric half-space and full-space filling with relative permittivity of $\varepsilon_{r}$, the corresponding FSS resonant frequency will be reduced to $f_{0}/\sqrt{(\varepsilon_{r}+1)/2}$ and $f_{0}/\sqrt{\varepsilon_{r}}$, respectively. $f_{0}$ is the resonant frequency of the free-space FSS without dielectric load.
(4) Considering the influence of the polarization mode and the oblique incident angle, multi-layer dielectric loading cascade and changing the shape of the metal patches are usually used to ensure that the polarization and angle insensitivity characteristics of FSS are within the acceptable range. However, there is an influence of inter-layer coupling factors when using dielectric loading cascade FSS. The calculation of the transmission matrix is computationally intensive and complex. In this paper, an MOPSO algorithm is used to omit the calculation of inter-layer coupling [27]. It compensates for the influence of medium, polarization, and incident angle on the frequency response in the original FSS design.

## 3. Implementation of the Design Method

This section verifies the effectiveness of the multi-layer FSS design method proposed in this paper by using a dual band-stop FSS and a triple band-pass FSS as examples.

### 3.1. Design of Dual Band-Stop FSS

When designing a dual band-stop FSS, two monolayer models are usually cascaded [34]. According to the design goal, we loaded two monolayer FSS models to generate two single band-stop responses and cascaded them to generate the desired dual band-stop responses [35,36].

**Figure 3:**
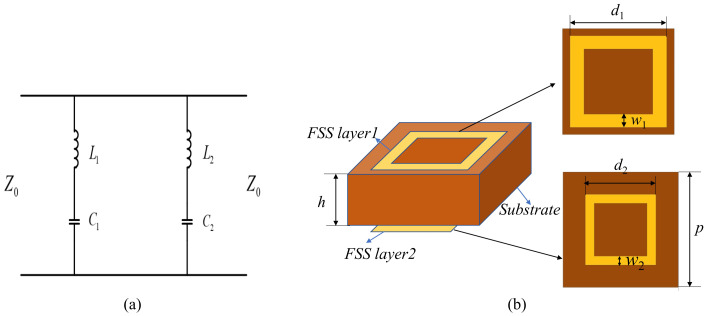
(**a**) The EC of the dual band-stop FSS. (**b**) Schematic diagram of the dual band-stop FSS.

Let the design target be a dual band-stop FSS. The center frequencies of the dual band-stop are $f_{1}=10$ GHz, $f_{2}=33.5$ GHz, covering the ultra-wideband from X to Ka-band. According to the design goals, a resonant circuit that meets the characteristics of FSS is constructed. Therefore, two sets of series-connected inductors and capacitors can be cascaded to achieve the goal. The values of $L_{1}$, $C_{1}$, $L_{2}$, and $C_{2}$ in Figure 3a are equal to 1.016 nH, 0.022 pF, 2.76 nH and 0.094 pF, respectively. Metal square loops can be cascaded to design an FSS that meets the above conditions.

According to Section 2, the structural parameters of FSS can be approximately calculated, as shown in Table 1. Figure 3b is the schematic diagram of the dual band-stop FSS structure.

**Figure 4:**
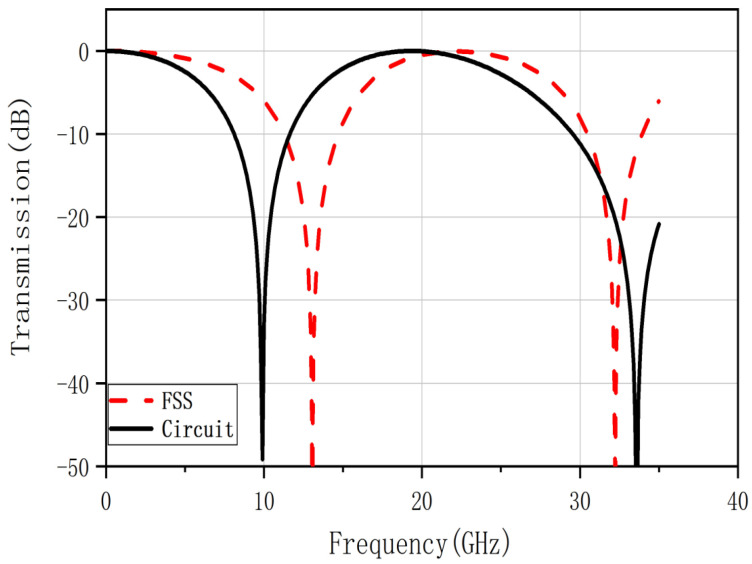
Model of the proposed dual band-stop FSS.

The transmission coefficient results of the FSS and the EC are shown in Figure 4. It is not consistent with the frequency response of the FSS model. The reasons for this situation are as follows:

(1) In the quantitative analysis, the complexity of the mathematical formulae, which omit some long and tedious factors in the calculation but have little impact on the overall count, may cause cumulative errors.
(2) The FSS and the resonant circuit on the transmission line are approximately equivalent. It only reflects that the two are roughly the same in terms of filtering property and are not precisely identical.
(3) As mentioned in the previous section, the dielectric substrate will change the operating frequency of the FSS during the design process.

It is common for the design concept to obtain the initial structure based on the target frequency response and existing research results in the field of expertise. The EM analysis of the FSS structure will be carried out using the EM simulation software, and the parameters scanned will be used to guide the modification of the structure parameters according to the parameter scan results. The disadvantage of this approach is that it is very time consuming and may not lead to the desired outcome. MOPSO offers a solution to this challenge.

In general, the multi-objective problem designs can be mathematically described as:

(7) $$\left\{\begin{array}{c} minF\left(X\right)={\left(f_{1}\left(x\right),f_{2}\left(x\right),\ldots f_{n}\left(x\right)\right)}^{T}\\ s.t.x\in X \end{array}\right.$$

where $x=x_{1},x_{2},\ldots ,x_{n}$ represents a vector of *n* designable parameters shaping a specific FSS, and X represents the space within which the parameters are designed. The *i*-th design objective is represented by $f_{i}\left(x\right),i=1,2,\ldots ,m$ and F(x) contains all of the design objectives. According to the concept of multi-objective programming, no objective can be completely superior to another. In other words, there is no *x* and *y*, and for all design goals, there exists $f_{i}\left(x\right)<f_{i}\left(y\right)$. Pareto dominance relations ≺ are defined as follows: for any two solutions x and y, if $f_{p}\left(x\right)\leq f_{p}\left(y\right)$ for all p=1,2,…,n and at least one design goal exists in order to make $f_{p}\left(x\right)<f_{p}\left(y\right)$, then *x* dominates *y*, i.e., x≺y. Thus, for any solution *x* included in the Pareto-optimal set, y∈X and y≺x are impossible.

The performance of FSS is mainly expressed in two aspects: the bandwidth of the band-pass or band-stop, and the stability of the transmission coefficient corresponding to the wave incidence angle. In this paper, our design goal is to obtain FSS structure parameters that are consistent with the frequency response of the equivalent circuit. As a result, the two objective functions of the MOPSO algorithm correspond to the root mean square error of the frequency response of the designed FSS from the equivalent circuit at the corresponding sampling points. The objective function of MOPSO is designed in the following:

(8a) $$\begin{array}{c} Obj_{1}=min\sqrt{\frac{1}{n}\sum_{i=1}^{n}{\left(S_{21}\left(i\right)-S_{21sim}\left(i\right)\right)}^{2}} \end{array}$$

(8b) $$\begin{array}{c} Obj_{2}=min\sqrt{\frac{1}{m}\sum_{j=1}^{m}{\left(S_{21}^{*}\left(j\right)-S_{21sim}^{*}\left(j\right)\right)}^{2}} \end{array}$$

where $S_{21}\left(i\right)$ and $S_{21sim}\left(i\right)$ denote the desired and the simulated transmission coefficients to the *i*-th sampling point when $S_{21sim}\left(i\right)\leq -10$ dB, respectively. $S_{21}^{*}\left(j\right)$ and $S_{21sim}^{*}\left(j\right)$ denote the desired and the simulated transmission coefficients to the *j*-th sampling point when $S_{21sim}^{*}\left(j\right)>-10$ dB. The number of samples in the corresponding frequency bands is denoted by *n* and *m*, respectively. For a population of *N*, to determine whether the *k*-th particle is a non-inferior solution, it must satisfy the following condition when compared to the other particles *n*:

(9) $$Obj_{1}\left(k\right)\leq Obj_{1}\left(n\right)\&\&Obj_{2}\left(k\right)<Obj_{2}\left(n\right)\|Obj_{1}\left(k\right)<Obj_{1}\left(n\right)\&\&Obj_{2}\left(k\right)\leq Obj_{2}\left(n\right)$$

Thus, the *i*-th particle provides a non-inferior solution. All non-inferior solutions currently form a set of non-inferior solutions.

The objective function depends on the error between the desired and the simulated transmission coefficients. The smaller objective value means that the frequency response corresponding to the proposed FSS is better fitted to the target value. For building blocks mentioned in this paper, the design problem can be phrased in terms of a multi-objective optimization problem with multiple dimensions and continuous optimization, the number of dimensions being determined by the structural parameters. Instead of a fully chance-oriented trial-error approach, the MOSPSO-based design approach systematically utilizes the MOPSO algorithm for multidimensional search operations.

The PSO algorithm has many similarities with other evolutionary computational techniques such as genetic algorithms. To verify the validity of MOPSO, the classical algorithm in GA, NSGAII, is adopted for comparison [37]. The parameters set of MOPSO is as follows: the number of populations is 40, the size of the repository is 50, the maximum number of iterations is 50, the initial weight is 0.5, the weight decay factor is set to 0.99, and acceleration factors are equal to 1 and 2, respectively. The NSGAII value parameters are set with the same number of populations and the mum number of iterations as MOPSO, the crossover probability is 0.9, and the variance probability is 0.1.

As shown in Table 2, the MOPSO algorithm is able to reduce the number of EM simulations. For the same population size and number of iterations, the MOPSO algorithm takes less time to optimize. The optimization efficiency is improved by about 18% compared with the NSGAII algorithm.

The upper and lower thresholds of the optimization parameters are determined by manufacturing errors. Their search ranges are set as follows: $d_{1}\in \left[5,6\right]$, $w_{1}\in \left[0.2,0.3\right]$, $d_{2}\in \left[2.5,3.5\right]$, $w_{2}\in \left[0.4,0.8\right]$. As shown in Table 3, both the MOPSO algorithm and the NSGAII algorithm can improve the accuracy of the model. Compared with the results obtained with parameter scanning in the CST software, the optimization algorithm can obtain the model structure parameters more quickly and accurately.

**Figure 5:**
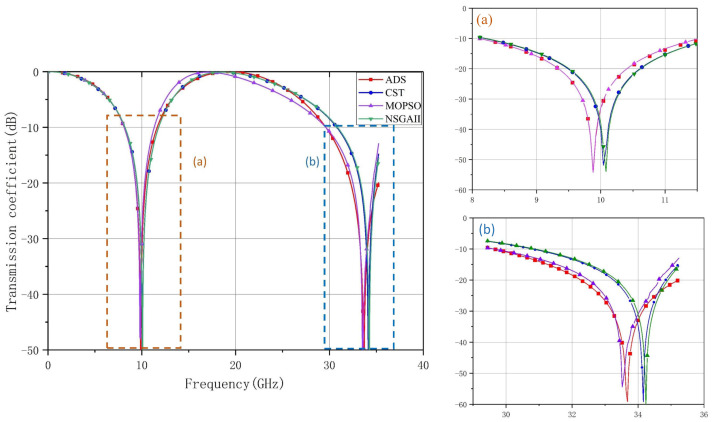
Comparison of transmission coefficients obtained from MOPSO, NSGAII, CST parameter scan optimization, and ECM in ADS. (**a**) Transmission coefficient in the frequency range of 8–11 GHz, (**b**) Transmission coefficient in the frequency range of 28.5–35 GHz.

The structural parameters of the proposed dual band-stop FSS optimized by different methods are shown in Table 3. As shown in Figure 5, the results obtained by MOPSO optimization are basically consistent with the results of EC. The NSGAII has the second-best effect, and the results of parameter scanning with CST software have a larger error than the results of EC. This is because the CST software can only set fixed compensation iterations for the parameters for EM simulation, which has a larger error. In contrast, MOPSO and NSGAII are global scans in the range of structural parameters, which make it easier to obtain optimal solutions.

### 3.2. Design of Triple Band-Pass FSS

**Figure 6:**
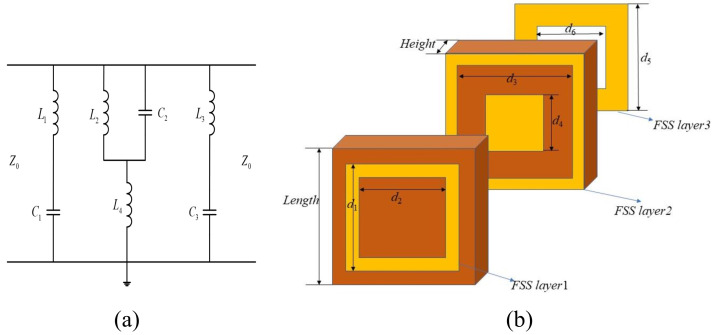
(**a**) The EC of the triple band-pass FSS. (**b**) Schematic diagram of the triple band-pass FSS.

This section introduces the design of a triple band-pass FSS loaded with a multi-layer dielectric. According to the filter circuit, this scheme works in the C-band, X-band, Ku-band, and the center frequencies are $f_{1}=4.53$ GHz, $f_{2}=8.52$ GHz, and $f_{3}=13.73$ GHz. The corresponding resonant circuit is constructed according to the design requirements, as shown in Figure 6a. The square loops and square slots cascade structure with a dielectric constant of 6 is used to design the triple band-pass FSS. The schematic diagram of the structure is shown in Figure 6b.

According to the design goals, the component values of the triple band-pass filter circuit can be received, as shown in Table 4.

**Figure 7:**
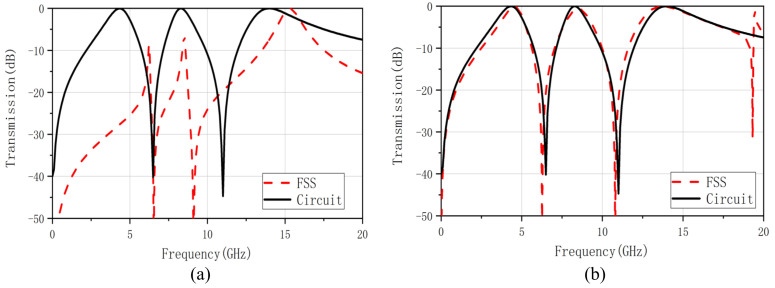
(**a**) Transmission coefficient of triple band–pass FSS for the ECM (shown in red) and desired (shown in black), (**b**) Transmission coefficient of optimized triple band–pass FSS for the optimized structure parameters by MOPSO algorithm (shown in red) and desired (shown in black).

From the corresponding relationship between FSS building blocks and the EC, the structural parameters obtained are shown in Table 5 below:

Figure 7a shows the transmission coefficient results of FSS constructed according to the structural parameters in Table 5. It shows that the frequency domain response of the designed FSS, although meeting the design requirements of the triple bands, is different from the frequency domain response of the circuit. It is mainly manifested in the center operating frequency and bandwidth. The 3 dB band-pass is not formed at the first two center frequencies, and the center frequency of the third band-pass is out of position. Relying on the EC to calculate the FSS structure parameters, there are substantial errors and uncertainties. For the unrealized band-pass and center frequency offset problems, the optimization goal needs to be more restricted when optimizing the designed FSS.

It is not only necessary to optimize the center frequencies but also to improve the bandwidth [38]. The objective functions of optimizing the working bandwidth are defined as Equation (8). $S_{21}$ and $S_{21sim}$ denote the desired and the simulated transmission coefficient when $S_{21sim}\geq -3$ dB, respectively. $S_{21}^{*}$ and $S_{21sim}^{*}$ denote the desired and the simulated transmission coefficient when $S_{21sim}^{*}<-3$ dB. The structural parameters that need to be optimized are $d_{1}$, $d_{2}$, $d_{4}$, $d_{6}$, among which $d_{1}\in \left(4,5\right)$, $d_{2}\in \left(3,6\right)$, $d_{4}\in \left(2,3\right)$, $d_{6}\in \left(3,4\right)$.

According to the MOPSO algorithm described in the previous section, optimize the structural parameters in Table 4. The optimized structural parameters are $d_{1}=5.00$ mm, $d_{2}=5.80$ mm, $d_{4}=3.00$ mm, $d_{6}=3.60$ mm. Figure 7b demonstrates the transmission coefficient of the optimized FSS and filter circuit. After the adjustment, the difference between the transmission frequency response of the FSS and the EC becomes smaller. The transmission coefficient results curve also coincides better. It can be seen that the design method combining empirical formula and optimization algorithm can make up for the deficiency of using the EC alone for the reverse deduction.

Based on the analysis in Section 2, it appears that the incidence angle is one of the most important indicators of the accuracy of the EC model and the practicality of the method proposed. For the purpose of verifying the precision, stability, and applicability of the FSS designed using the EC method, we analyzed the transmission coefficients under both TE and TM polarizations.

**Figure 8:**
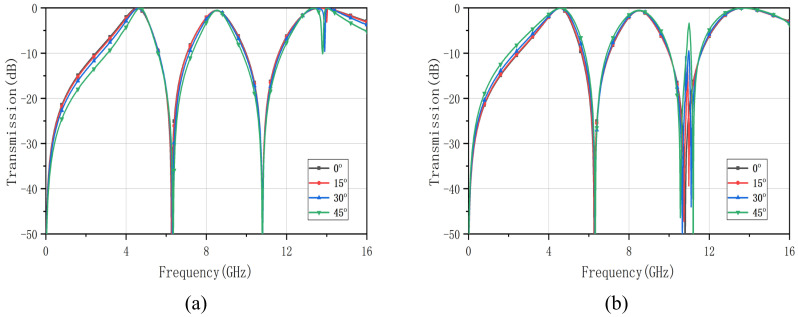
Transmission coefficients under different oblique incidence angles of the optimized triple band–pass FSS. (**a**) TE polarization, (**b**) TM polarization.

Figure 8 reflects the polarization and angular stability of the FSS under different polarization and oblique incidence conditions. Under TE and TM polarization, the triple band-pass FSS at oblique incidence has good stability in the first two bands with a maximum oblique incidence angle of up to 45${}^{\circ }$.

**Figure 9:**
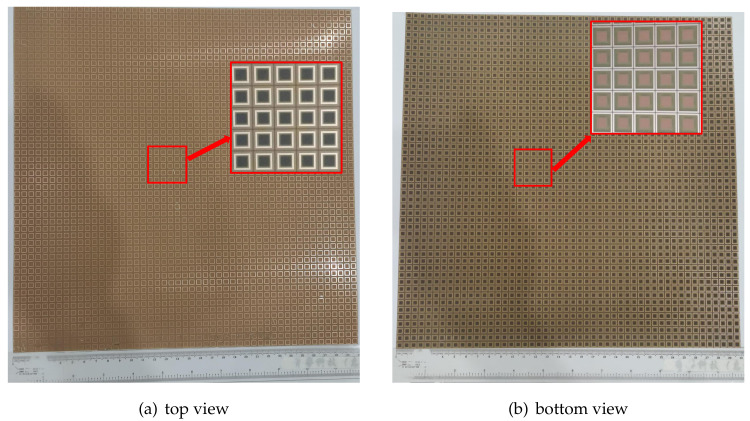
Photos of the triple band-pass FSS fabricated prototype.

To verify the effectiveness of the EC-based FSS design scheme proposed in this paper, a triple band-pass FSS is fabricated and measured as an example. To simulate the infinite plane as much as possible, the final object has a size of the final object is 300 mm × 300 mm, containing 50 × 50 building blocks, as shown in Figure 9.

**Figure 10:**
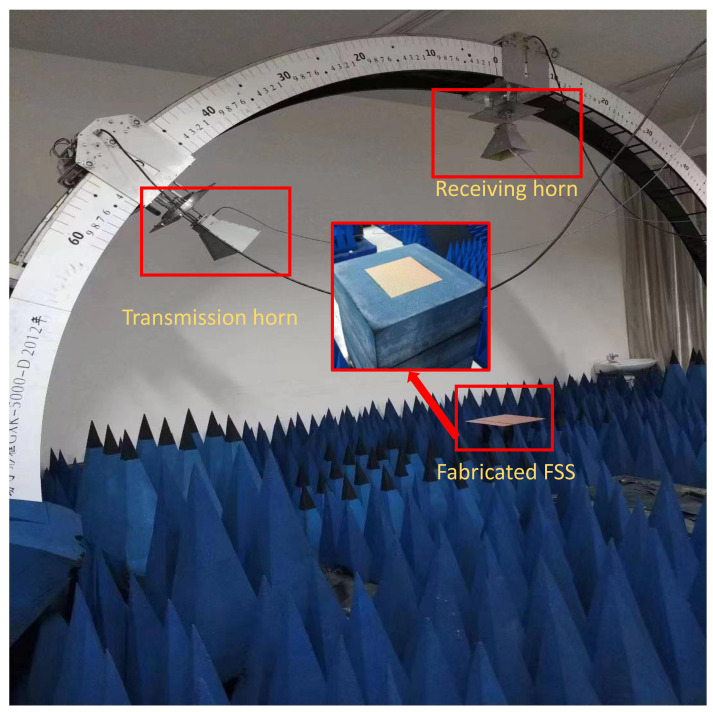
The arch measurement setup of the fabricated FSS. The transmission and receiving horns are mounted on an arc perpendicular to the plane of the fabricated FSS.

Figure 10 shows the schematic of the FSS measurement setup. The measurement approach employs the arch method. Both the transmission and receiving horns are mounted on an arc perpendicular to the plane of the fabricated FSS located. FSS is excited by the transmission horn, and its reflected signal is detected by the receiving horn. To ensure the test accuracy, a metal plate with identical dimensions as the triple band-pass FSS to be tested is prepared for calibration. The panel is surrounded by absorbers to reduce diffractions from the edges of the FSS panel. The antenna is located approximately 2 m away from the transmitting and receiving antennas to ensure that both the transmitter and receiver are in the far-field region.

**Figure 11:**
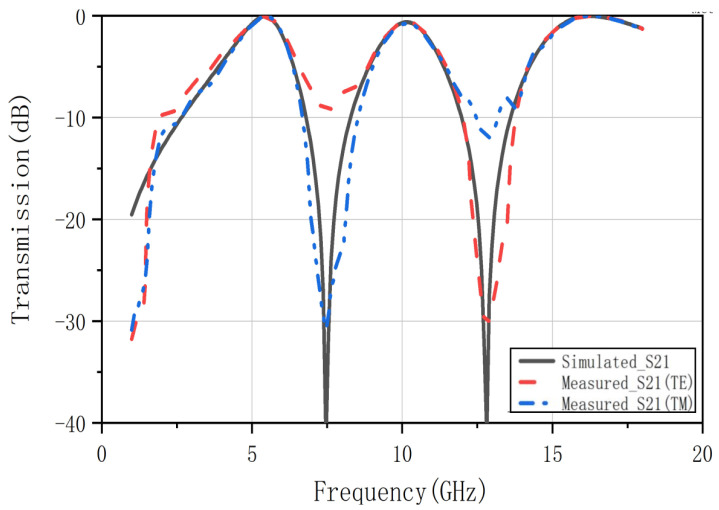
Comparison of transmission coefficient results between full-wave simulation and measurement data.

Figure 11 shows the actual measurement results of the triple band-pass FSS. It can be seen that under TE polarization and TM polarization, the actual measurement results are in good agreement with the simulation results. Therefore, the actual performance of the designed FSS meets the requirements and also proves that the method proposed in this paper is effective.

## 4. Conclusions

An EC-assisted MOPSO method to accelerate the reverse design of the FSS based on the building blocks is proposed in this paper. In place of entirely relying on empirical formulas and precise calculations, the proposed method performs the initial design based on a correspondence between the building blocks and the EC. Following that, the MOPSO algorithm is used to optimize the structural parameters of the building blocks in order to achieve the exact design. By illustrating dual band-stop and triple band-pass FSS with numerical and measurement results, the proposed method is shown to be valid and accurate. Based on the simulation results, it appears that this method offers good consistency in frequency response for FSSs and filter circuits designed using this method. Furthermore, it has been shown that the triple band-pass FSS frequency performance maintains its stability under a wide range of oblique incident angles up to 45° in both TE and TM polarization. With the method presented in this paper, the inverse design process of multi-layer FSSs is simplified and the complexity of manufacturing is reduced.

## Figures and Tables

**Table 1:** Structure parameters of the dual band-stop FSS (unit: mm).

*h*	*p*	$d_{1}$	$w_{1}$	$d_{2}$	$w_{2}$
0.25	6	5	0.24	2.25	0.1

**Table 2:** Comparison of computational cost.

Optimization Approach	Numbers of EM Simulation	CPU Time/h	
		Total	Relative (%)
MOPSO	2040	40.78	82.01%
NSGAII	2200	60.70	100%

**Table 3:** FSS structure parameters for different optimization approaches (unit: mm).

Parameters	Optimization Approach		
	Parameter Sweep	MOPSO	NSGAII
$d_{1}$	5.5	5.50	5.49
$w_{1}$	0.25	0.21	0.20
$d_{2}$	3	3.28	3.00
$w_{2}$	0.5	0.62	0.50

**Table 4:** The EC parameters value of triple band-pass FSS.

$L_{1}$	$C_{1}$	$L_{2}$	$C_{2}$	$L_{3}$	$C_{3}$	$L_{4}$
0.06 nH	0.1 pF	1.22 nH	0.57 pF	5.1 nH	0.03 pF	2.12 nH

**Table 5:** FSS structure parameters (unit: mm).

Length	Height	$d_{1}$	$d_{2}$	$d_{3}$	$d_{4}$	$d_{5}$	$d_{6}$
6	0.25	4.3	3	5.8	2.5	4.5	3.5

## Data Availability

Not applicable.
